# Rap1 and Rap2 Antagonistically Control Endothelial Barrier Resistance

**DOI:** 10.1371/journal.pone.0057903

**Published:** 2013-02-28

**Authors:** Willem-Jan Pannekoek, Jelena R. Linnemann, Patricia M. Brouwer, Johannes L. Bos, Holger Rehmann

**Affiliations:** Molecular Cancer Research, Centre of Biomedical Genetics and Cancer Genomics Centre, University Medical Center Utrecht, Utrecht, The Netherlands; Northwestern University Feinberg School of Medicine, United States of America

## Abstract

Rap1 and Rap2 are closely related proteins of the Ras family of small G-proteins. Rap1 is well known to regulate cell-cell adhesion. Here, we have analysed the effect of Rap-mediated signalling on endothelial permeability using electrical impedance measurements of HUVEC monolayers and subsequent determination of the barrier resistance, which is a measure for the ease with which ions can pass cell junctions. In line with its well-established effect on cell-cell junctions, depletion of Rap1 decreases, whereas activation of Rap1 increases barrier resistance. Despite its high sequence homology with Rap1, depletion of Rap2 has an opposite, enhancing, effect on barrier resistance. This effect can be mimicked by depletion of the Rap2 specific activator RasGEF1C and the Rap2 effector MAP4K4, establishing Rap2 signalling as an independent pathway controlling barrier resistance. As simultaneous depletion or activation of both Rap1 and Rap2 results in a barrier resistance comparable to control cells, Rap1 and Rap2 control barrier resistance in a reciprocal manner. This Rap1-antagonizing effect of Rap2 is established independent of junctional actin formation. These data establish that endothelial barrier resistance is determined by the combined antagonistic actions of Rap1 and Rap2.

## Introduction

The endothelium is the cell layer that lines the circulatory system. Composed of specified endothelial cells that tightly anchor together, the endothelium forms a barrier that protects the underlying tissue from substances in the blood. At the same time, it must allow the passage of fluids, ions and immune cells upon request. Hence, the permeability of the endothelium is tightly and dynamically regulated [1]. The focal point of regulation is the Adherens Junction, at which VE-cadherin proteins interact to anchor neighbouring cells. Intracellularly, VE-cadherin interacts with many regulatory proteins, amongst which are α- and β-catenin that link VE-cadherin to the actin cytoskeleton, thereby conferring monolayer rigidity. Binding of p120-catenin to VE-cadherin prevents VE-cadherin endocytosis to facilitate cell-cell adhesion. Agents that induce permeability are well known to impinge on VE-cadherin and catenin proteins [2].

Tightening of endothelial junctions is induced by hormones and agonists that generally induce the second messenger cAMP [3]–[5]. Epac1 is one of the targets of cAMP that functions in endothelial cell-cell adhesion [6]–[9]. Epac1 decreases permeability via its guanine nucleotide exchange factor (GEF) activity towards the Rap1 G-proteins [10], as well as through direct effects on microtubules [11]. Rap1, which occurs as two isoforms termed Rap1A and Rap1B, is a critical regulator of cell-cell junctions [12]. Rap1 controls endothelial permeability upon cAMP increase, but basal levels of permeability also depend on Rap1, to which end it is constitutively activated mainly by PDZ-GEF [13]. Both basal and cAMP-induced effects of Rap1 are predominantly relayed by the Rap1A isoform [13], [14]. Apart from dynamic activation by GEFs, Rap1 activity can be regulated by GAPs, which catalyze the hydrolysis of GTP to inactivate G-proteins [15]. Overexpression of RapGAPs is generally used to abolish Rap1 activity, resulting in for instance impaired epithelial cell-cell junction formation [16], [17] and increased endothelial permeability [7], [9]. One report has investigated the endogenous role of RapGAPs in cell-cell adhesion. Here, stable depletion of RapGAP1 actually prevents the formation of Adherens Junctions between carcinoma cells [18]. Downstream of Rap1 several effects have been observed, which include actin reorganization, actin mediated stabilization of VE-cadherin, Rac1 activation, KRIT mediated enrichment of junctional β-catenin and KRIT mediated downregulation of tension [6]–[8], [19]–[22].

Despite the large body of data on Rap1 in the control of endothelial permeability, the function of Rap2 in this process has not been explored. Rap2 exists as three isoforms, termed Rap2A, Rap2B and Rap2C [23]–[25]. Most sequence differences within the Rap family reside in the C-terminal part of the proteins, which generally determines subcellular localization of Ras-like G-proteins [26]. The RapGEFs Epac and PDZ-GEF activate both Rap1 and Rap2 [27]–[29], whereas C3G and RasGEF1 show specificity towards Rap1 and Rap2, respectively [30], [31]. Rap1 and Rap2 both bind effector proteins containing an RA domain and to date no RA-domain containing proteins that specifically bind either Rap1 or Rap2 have been reported. Rap2, however, does also bind to the citron homology (CNH) domain of TNIK, MINK and MAP4K4, which together form the GCK-IV subgroup of Ste20 kinases that function in MAPK signaling and are involved in many diverse signaling pathways, amongst which is severing of the actin cytoskeleton [32]–[35]. Given the important role of the actin cytoskeleton in cell-cell adhesion dynamics, these proteins are likely to function here as well. Indeed, overexpression of MINK decreases junctional staining of β-catenin in MCF7 cells [36]. During mouse gastrulation, MAP4K4 activates p38 to induce downregulation of E-cadherin and concomitant EMT [37].

Here we studied the role of Rap proteins on the permeability of monolayers of Human umbilical vein endothelial cells (HUVEC) using Electrical Cell Substrate Impedance Sensing (ECIS) to measure the impedance, which is opposed to an alternating electrical current of a given frequency by a monolayer of cells grown on an electrode. Several groups have correlated Rap signalling to an increase in the absolute value of the impedance or the real part of the impedance (then interpreted as the resistance of a serial RC-element) [9], [11], [13], [14], [38], [39]. This macroscopic measure describes the general properties of the cell-covered electrode. Lo et al., have developed a physical model which describes the frequency dependency of the impedance by the three constants α, C_m_ and R_b_ [40]. These constants are originated in the microscopic properties of the cell monolayer. α depends on the diameter of the cells, the distance between electrode surface and cell, and the electrical conductivity of the tissue culture medium. C_m_ is the capacity of the cell membrane. R_b_, abbreviating barrier resistance, describes the resistance, which is opposed to the current when passing the junctions between the cells. As electrical current in solution is driven by moving ions, the barrier resistance is a measure of the ease at which ions can pass cell-cell junctions and thus a direct measure of junctional permeability.

Using ECIS we find that, in contrast to the well-established barrier resistance-inducing function of Rap1, depletion of Rap2 results in an increase in barrier resistance. The Rap2 effects depends on a signalling module composed of the Rap2 specific GEF RasGEF1 and the Rap2 effector MAP4K4. Importantly, Rap1 and Rap2 control barrier resistance in a reciprocal manner, as simultaneous depletion of both Rap1 and Rap2 or simultaneous activation of Rap1 and Rap2 by RapGAP depletion results a barrier resistance comparable to control cells. Furthermore, we find that the Rap1-antagonizing effect of Rap2 is established independent of junctional actin formation.

## Materials and Methods

### Reagents and Antibodies

007-AM (8-pCPT-2'-O-Me-cAMP-AM) was from Biolog Life Sciences (Bremen, Germany) and used at a concentration of 1 µM [41]. The Rap1 antibody was from Santa Cruz. Antibodies against Rap2, Spa1, VE-cadherin and β-catenin were from BD Bioscience and the α-Tubulin antibody was from Calbiochem. The V5 antibody, fluorescently labeled Phalloidin and secondary antibodies were from Invitrogen.

### Cell Culture and Transfections

HEK293T cells (ATCC), used for production of lentivirus, were cultured at 37°C and 6% CO_2_ in Dulbecco’s Modified Eagle Medium supplemented with 10% fetal bovine serum, 2 mM L-glutamine and antibiotics. Human Umbilical Vein Endothelial Cells (HUVECs) (Lonza) were grown at 37°C and 6% CO_2_ on tissue culture dishes coated with 0.5% Gelatin in EBM-2 culture medium (Lonza) supplemented with EGM-2 SingleQuots (EGF, hydrocortisone, fetal bovine serum, VEGF, FGF-B, R3-IGF-1, ascorbic acid, GA-100, heparin) (Lonza). HUVECs were cultured maximally 14 days before experiments. siRNA transfections were performed 72 hours before experiments with 50 nM ON-TARGETplus SMARTpools (Dharmacon Inc.) targeting indicated genes using Dharmafect-1 (Dharmacon Inc.). When multiple genes (up to five) were targeted simultaneously the total concentration of the pooled SMARTpools was kept at 50 nM. Overexpression of proteins in HUVECs was established by lentiviral transduction. To this end, pLV-CMV-bc-GFP (kindly provided by Patrick Derksen, Department of Pathology, University Medical Center Utrecht) was modified by inserting an N-terminal V5-tag by site-directed mutagenesis and a Gateway Cassette into the Mung Bean Nuclease-blunted Sal1 site. Next, V12Rap1A was inserted using the Gateway system (Invitrogen) to yield pLV-CMV-V5-V12Rap1A-bc-GFP. For lentivirus production, the growth medium of HEK293T cells was replaced by EBM-2 growth medium, upon which these cells were transfected using FuGene6 (Roche) with the appropriate lentiviral expression plasmid or an empty vector control together with third-generation packaging constructs. HUVECs were infected 48 hours before experiments using the undiluted growth medium of virus-producing HEK293T cells supplemented with 8 mg/l polybrene.

### ECIS

**Figure 1:**
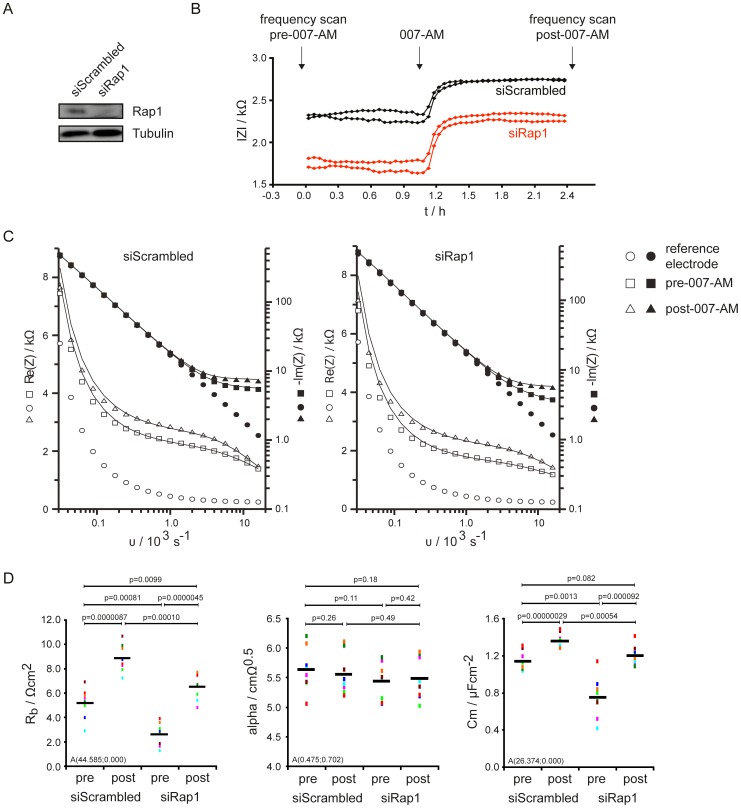
Rap1 controls the barrier resistance. HUVEC monolayers transfected with siScrambled or siRap1 were grown on electrodes and analyzed by ECIS. (A) Western blot shows Rap1 depletion efficiency. (B) Impedance was recorded over time at 4000 Hz (here plotted as absolute value). (C) Frequency scans were performed before (pre) and after (post) stimulation with 007-AM as indicated in Fig. 1B. The real part of the impedance is displayed on the right axis (open symbols) and the imaginary part on the left axis (closed symbols). A single electrode of siScrambled (left) and siRap right is shown. The data were fitted (shown as lines) in relation to the reference electrode by the model of Lo et al. (D) The obtained values for the barrier resistance (R_b_), α and C_m_ are represented in bar diagrams. Different colors represent individual independent experiments (n = 8). Averages are indicated by black lines.

ECIS measurements were essentially performed as described previously [13]. 48 hours after siRNA transfection and/or 24 hours after lentiviral infection, HUVECs were plated onto L-cysteine reduced, Fibronectin-coated 8W10E electrodes (Applied Biophysics) at a density of 1×10^5^ cells/well and grown to confluency for another 24 hours. The time-dependent impedance was measured at 37°C and 6% CO_2_ using a 1600R Electrical Cell Impedance Sensing (ECIS) system (Applied Biophysics) at 4000 Hz. Frequency scans were performed before and after the timelapse recording, as indicated in figure 1B. These frequency scans were used to calculate α, R_b_ and C_m_ with ECIS software (v1.2.50.0 PC) from Applied Biophysics. For this analysis frequencies were limited to the range from 62.5 Hz to 16000 Hz, as a strong inductive component was observed at higher frequencies, which can not be processed by the used algorithms. Each data point in the graph represents the average of individual electrodes (N = 4) within an experiment. Multiple independent experiments are shown using differently colored data points. Averages are indicated by a black line. Statistical analysis of whole data set variance was performed by One-way ANOVA, which is depicted as A(F-score;P-value). Statistical analysis of variance between two groups within one experiment was calculated by Student’s t-test (two-tailed, paired).

### QPCR

8×10^5^ HUVEC cells were plated 48 hours after siRNA transfection onto Fibronectin-coated 6 cm dishes and grown for another 24 hours. Total RNA was isolated using the RNeasy Mini Kit (Qiagen) and transcribed into cDNA using the iScript cDNA Synthesis Kit (BIO-RAD). Rap2 isoform expression was quantified by SYBR green real-time PCR on a C1000 Thermal Cycler (BIO-RAD) using the following primers: CATGCTGTTCTGCATGTAAC (Rap2A-forward), CAAGTTCTGCAGTGGAGTAG (Rap2A-reverse), GACTGATTGCGATTCTGAGG (Rap2B-forward), CACACTGTATTGGCATCAGT (Rap2B-reverse), CAGGATATCAAGCCAATGAG (Rap2C-forward) and CTGAAGACATAACCTCTCTTTC (Rap2C-reverse). Nonspecific signals were excluded based on non-template control samples. Expression levels of each gene were monitored relative to the HPRT gene as difference in cycle number (ΔC). To allow the comparison of independent experiments, an identical reference sample of untreated cells were included in each experiments repetitively. Comparison was performed by calculating the differences to the reference sample ΔΔC. Statistical analysis was performed on the averaged ΔΔC values by One-way ANOVA, which is depicted as A(F-score;P-value). In addition statistical analysis of variance between two groups within one experiment was performed by Student’s t-test (two-tailed, unpaired).

### Immunofluorescence

48 hours after siRNA transfection, HUVECs were plated onto Fibronectin-coated glass coverslips in 24-well plates (2×10^5^ cells/well) and grown to confluency for another 24 hours. After 10 minutes stimulation with 1 µM 007-AM, cells were fixed with 4% formaldehyde for 20 minutes, permeabilized with 0.1% TX-100 for 3 minutes and blocked with 1% BSA for at least 2 hours. Next, cells were incubated with indicated primary antibodies for 1 hour, secondary antibody for 30 minutes and mounted onto glass slides, which were subsequently examined on an Axioskop 2 mot plus microscope (Zeiss) with a 40× immersion oil objective and Axiocam camera. To quantify junctional actin, corresponding pictures of β-catenin and phalloidin were simultaneously loaded into ImageJ, segmented by threshold and colocalization was calculated using the Colocalization Indices Plugin written by Kouichi Nakamura (available at http://www.mbs.med.kyoto-u.ac.jp/imagej/index.html). Graphs show averages of the Correlation Coefficients (t CC) of 5 pictures within an experiment as colored data points, were multiple independent experiments are represented by different colors. Averages are indicated by a black line. Statistical analysis of whole data set variance was performed by One-way ANOVA, which is depicted as A(F-score;P-value). Statistical analysis of variance between two groups within one experiment was calculated by Student’s t-test (two-tailed, paired).

## Results

To confirm that Rap1 affects the impedance of endothelial monolayers and establish which particular microscopic property is responsible for this effect, detailed ECIS analysis was applied to the direct effect of Rap1A and Rap1B, which were depleted by siRNA in HUVEC. In addition, Rap1 was activated by stimulation with 007-AM. 007-AM is a cAMP analogue that selectively activates Epac, a Guanine Nucleotide Exchange Factor (GEF) for Rap [10]. The stability of the monolayer was controlled by monitoring the impedance at 4000 Hz over time (Fig. 1 A, B). Frequency scans were recorded before and after the application of 007-AM as indicated in figure 1B and fitted in relation to a cell free reference electrode to the model of Lo et al. [40]. Good agreement between the data points and the calculated values was obtained (Fig. 1C). The obtained constants are summarised in Fig. 1D. Activation of Rap by 007-AM induced an approximate 1.5-fold increase in barrier resistance (R_b_), whereas depletion of Rap1 decreased the barrier resistance. A small effect on C_m_ was observed whereas α was not affected.

**Figure 2:**
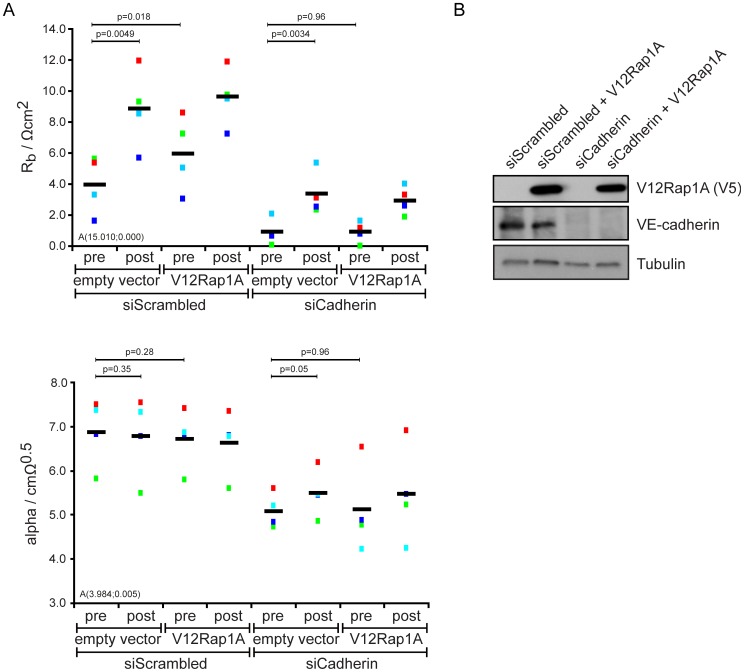
Rap1 controls the barrier resistance via Cadherins. (A) HUVECs transfected with siScrambled or both siVE-Cadherin and siP-cadherin were transduced with control or V12Rap1A containing lentivirus and analysed before (pre) and after (post) stimulation with 007-AM as in Figure 1. Different colours represent individual independent experiments (n = 4). Averages are indicated by black lines. (B) Total cell lysates of an experiment shown in A, subjected to SDS-page and subsequent Western blotting to show V12Rap1A and VE-cadherin protein expression.

To confirm that modulation of the barrier resistance by Rap1 reflects an effect of Rap1 on cell-cell junctions, VE-cadherin and P-cadherin were depleted from HUVEC. Under these conditions the barrier resistance was close to zero, whereas α was hardly affected (Fig. 2). C_m_ was strongly reduced, but variable, partially due to the limitation of data acquisition of frequencies below 40000 Hz (data not shown). Proper analysis requires an intact monolayer [40]. Depletion of Cadherins is approaching this limit as the barrier resistance was close to zero, but is not breaking this limit as α stayed hardly affected (Fig. 2). This confirmed the robustness of the model. Over-expression of the constitutively active V12Rap1A mutant was not able to rescue cadherin depletion (Fig. 2). In contrast to V12Rap1A overexpression, stimulation with 007-AM did induce a minor increase in the barrier resistance when Cadherins are depleted (Fig. 2). This barrier tightening effect of 007-AM was also apparent in monolayers transduced with V12Rap1A, possibly caused by incomplete transduction efficiency of V12Rap1A, remaining GEF sensitivity of the V12Rap1A, GEF activity towards endogenous Rap1 or the previously reported Rap1- and Cadherin-independent effect of Epac on permeability [11], [13]. In conclusion, ECIS analysis allows the direct correlation of Rap signalling to junctional permeability.

**Figure 3:**
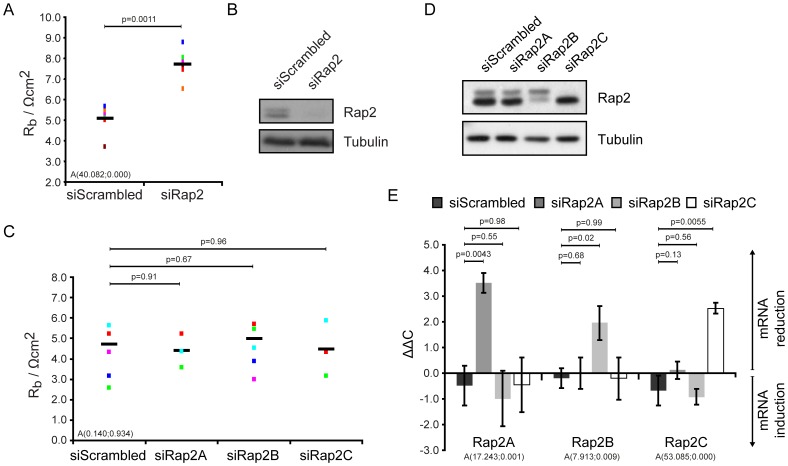
Rap2 decreased the barrier resistance. Simultaneous depletion of all three Rap2 isoforms (A, B) increases the barrier resistance of HUVEC monolayers, whereas individual depletion of Rap2A, Rap2B and Rap2C showed no effect (C, D). Analysis was performed as in Figure 1. Different colors represent individual independent experiments (A: n = 6, C: n = 3 for siRap2A and siRap2C, n = 6 for siRap2B). Averages are indicated by black lines. (B, D) The Western blots show Rap2 protein depletion. (E) HUVECs transfected with siScrambled, siRap2A, siRap2B or siRap2C were grown to confluency, after which RNA was extracted and Rap2 mRNA levels were assessed by QPCR. The histogram shows averages of three independent experiments which were related to one identical reference mRNA extraction of untreated cells. Error bars indicate standard deviation.

Whereas the effect of Rap1 on junctional permeability is extensively described, the function of the highly homologous Rap2 proteins in this process has remained unexplored. To analyse a putative contribution of Rap2A, Rap2B and Rap2C, these proteins were depleted from HUVEC and assayed for their effect on the barrier resistance (Fig. 3A, B). Depletion of the three Rap2 proteins increased the barrier resistance twofold. This is opposite to the effect of Rap1 depletion (Fig. 1) and indicates that Rap2 increases junctional permeability. Individual depletion of Rap2A, Rap2B or Rap2C resulted in no significant effect (Fig. 3C). On Western blots, a Rap2 antibody detected two bands which were predominantly sensitive to depletion of Rap2B (lower band) and Rap2C (upper band) (Fig. 3D). The expression of all three Rap2 isoforms was detected by RT-QPCR in HUVECs, but the used set-up did not allow determining and comparing absolute expression levels (Fig. 3E). The used RNAi oligos showed no significant effect on the respective non targeted isoforms (Fig. 3E). As the Rap2 antibody detected recombinant version of the three Rap2 isoforms equally well (data not shown), western blot analysis would suggests that Rap2B and Rap2C are the major isoforms in HUVECs (Fig. 3D) and act redundantly to control barrier resistance.

**Figure 4:**
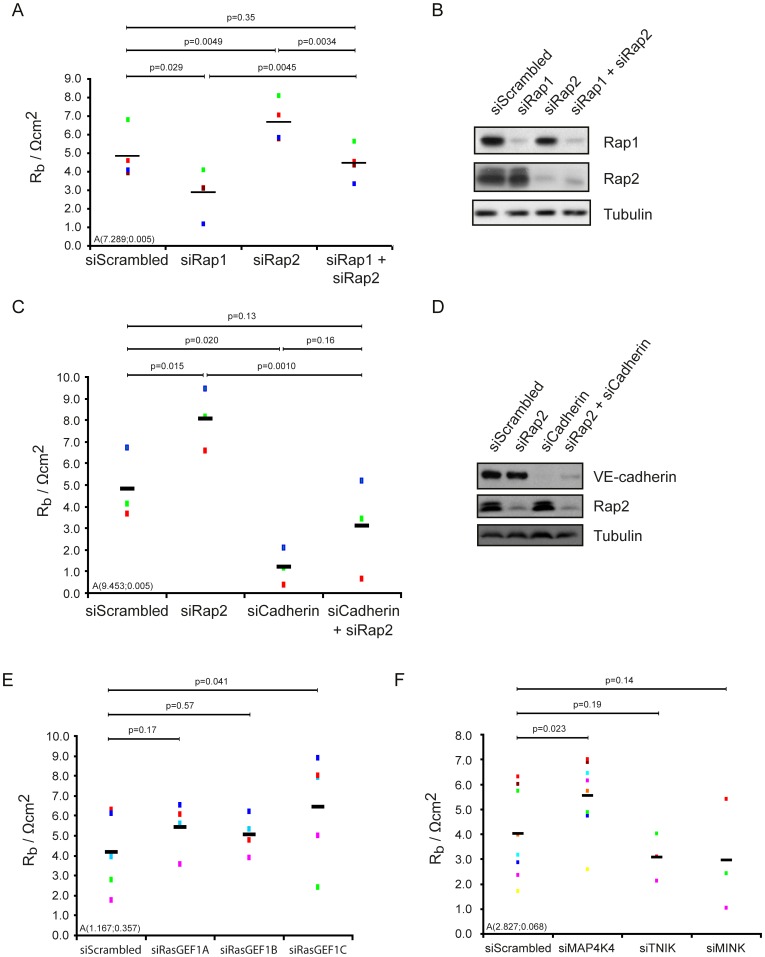
Rap2 antagonizes Rap1 by an own signalling module. (A, B) The barrier resistance of HUVECs transfected with siRNAs targeting the Rap1 proteins, the Rap2 proteins or all five Rap proteins was analysed. Different colors represent individual independent experiments (n = 4). Averages are indicated by black lines. (B) The Western blot shows Rap1 and Rap2 protein depletion. (C, D) The barrier resistance of HUVECs transfected with siRNAs targeting the Rap2 proteins, the Cadherins VE-cadherin and P-cadherin or all five proteins was analysed. Different colors represent individual independent experiments (n = 3). Averages are indicated by black lines. (D) The Western blot shows Rap2 and VE-cadherin protein depletion. (E) The barrier resistance of HUVECs transfected with siRNAs targeting RasGEF1A, RasGEF1B and RasGEF1C or (F) the Ste20 kinases MINK, TNIK or MAP4K4. All ECIS experiments were performed as in Figure 1. Different colors represent individual independent experiments (E: n = 4 for siRasGEF1A and siRasGEF1B, n = 5 for siRasGEF1C, F: n = 9 for siMAP4K4 and n = 3 for siTNIK and siMINK). Averages are indicated by black lines.

The observation that Rap2 depletion increased barrier resistance suggests that Rap1 and Rap2 can antagonize each other. Indeed, when HUVECs are depleted of all five Rap proteins, the barrier resistance was comparable to control levels (Fig. 4A, B). Similar to Rap1, the effect of Rap2 on the barrier resistance depends on Cadherins (Fig. 4C, D). In this context it is interesting to notice that RapGEFs regulating endothelial junctions, PDZ-GEF and Epac1, are activating Rap1 and Rap2 [27]–[29] and that many effectors of Rap1 interact with Rap2 as well [42]. Since Rap2 antagonizes the effect of Rap1 on barrier resistance, Rap2 should utilize at least partially its own unique GEFs and effectors. Specific activation of Rap2 proteins has been demonstrated for the RasGEF1 family of RapGEFs, which consists of three closely related proteins, two of which where characterised *in vitro* and found to display GEF activity towards Rap2 but not Rap1 [31]. Depletion of RasGEF1C in particular increased the barrier resistance (Fig. 4E). To identify a potential Rap2 effector protein, the members of the GCK-IV subgroup of Ste20 kinases were knocked down. Similar to depletion of Rap2 and RasGEF1, depletion of MAP4K4 caused a marked increase in barrier resistance (Fig. 4F). No effect of siMINK or siTNIK was observed. In HUVECs the RasGEF1 family and MAP4K4 therefore form a Rap2 specific signalling route, which mediates the Rap1 antagonising effect of Rap2.

**Figure 5:**
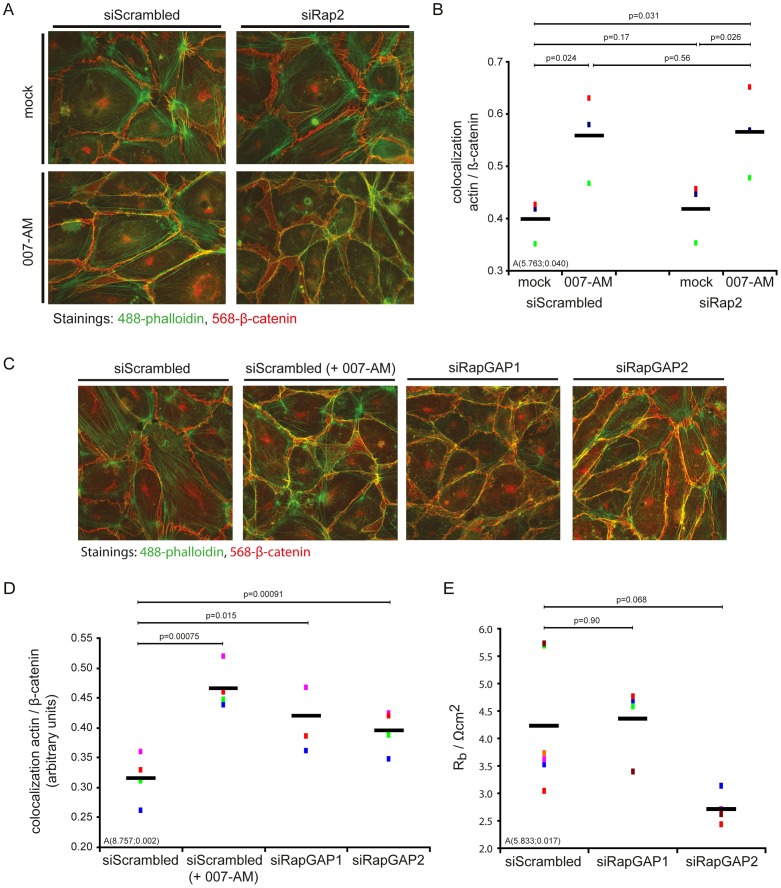
Rap2 antagonizes Rap1 downstream of junctional actin formation. HUVECs transfected with siRNAs targeting either all three Rap2 proteins (A, B) or the RapGAPs RapGAP1 or RapGAP2 (C, D) were plated onto coverslips, stimulated with or without 007-AM for 10 minutes and stained for F-actin (phalloidin, green) and β-catenin (red). Quantifications of the amount of signal correlation between β-catenin and F-actin (0 = random, 1 = perfect correlation) are shown in (B) and (D). Different colors represent individual independent experiments (B: n = 3, D: n = 4, E: n = 4 for siRapGAP1 and n = 5 for siRapGAP2). Averages are indicated by black lines. (E) The effect of RapGAP1 or RapGAP2 depletion on the barrier resistance was determined as described in Figure 1.

Rap1 has been suggested to regulate endothelial permeability by inducing junctional actin [7], [8], [11], [21]. Conversely, Rap2 has been proposed to disrupt filamentous actin via members of the GCK-IV subgroup of Ste20 kinases, which controls the activity of the actin-severing-enzyme Gelsolin [35], [43]. As Rap1 and Rap2 both impinge on Cadherins, this might occur by the opposite regulation of junctional actin formation. Indeed, HUVECs stained for F-actin and the junction marker β-catenin showed a transverse actin fiber pattern that changes to junctional actin upon activation of Rap1 by addition of 007-AM (Fig. 5A). Depletion of Rap2 did not affect junction morphology as β-catenin staining was indiscernible from control cells. Intriguingly, Rap2 depletion did not induce the typical junctional actin formation that would normally correlate with its permeability-reducing effect. Instead, actin fibres ran in transverse stress fibres, which did relocate to the cell-cell junction upon activation of Rap1 by addition of 007-AM, which was quantified to be similar to control cells (Fig. 5B). Hence, both basal and Rap1-induced actin morphology were similar in control and Rap2-depleted cells, suggesting that Rap2 does not utilize its actin-severing capacity to decrease the barrier resistance and therefore antagonizes Rap1 independent of junctional actin. To corroborate this result further, we hypothesized simultaneous activation of Rap1 and Rap2 to increase junctional actin while leaving the barrier resistance unaffected. Here, we were intrigued by a recent report showing that RapGAP is required for, rather than antagonizing, epithelial cell-cell adhesion, as these RapGAP-depleted epithelial cells show enhanced activation of both Rap1 and Rap2 [18]. Concomitant with its effect on Rap1 activity, depletion of either RapGAP1 or RapGAP2 strongly induced the formation of junctional actin (Fig. 5C, D). Nevertheless, depletion of RapGAP1 or RapGAP2 had no effect on the barrier resistance (Fig. 5E). This result suggests that Rap2 antagonizes Rap1 independent of junctional actin formation and that Rap1 activity and barrier resistance can be uncoupled by Rap2 activation. Furthermore, these data establish that the relative ratio between Rap1 and Rap2 activity controls the barrier resistance.

## Discussion

The Rap1 small G-protein is well established to be required for tightening of endothelial cell-cell junctions and concomitant junctional permeability by assessing binding of cells to Fc-VE-cadherin coated surfaces and monolayer permeability of mannitol, HRP or fluorescent dextran [8], [9], [20], [44]. Monolayer permeability can be followed in real time by determining the barrier resistance using electrical cell impedance sensing (ECIS). To date, interpretation of these data is restricted to the notion that Rap1 activity correlates with the impedance as a macroscopic property of the cell monolayer [9], [11], [13], [14], [38], [39]. These effects of Rap1 on impedance are attributed to cell-cell adhesion, although many biological processes affect this measure. Here, impedance measurements are connected to microscopic properties of the monolayer by analysing the data based on the physical model of Lo et al [40]. Rap proteins have a clear effect on the barrier resistance, which is determined by paracellular ion flow only and therefore a measure of junctional permeability.

The reciprocal effects of Rap1 and Rap2 might seem rather surprising, as the RapGEFs PDZ-GEF and Epac1 activate both G-proteins *in vitro* and *in vivo* [27]–[29]. Indeed, Rap2 is activated in HUVECs by Epac1 upon stimulation with 007-AM (data not shown), although it remains to be established whether the Epac-activated pool of Rap2 affects the barrier resistance. On the other hand, opposite effects of Rap1 and Rap2 on neurite morphology have been clearly established [45]–[50]. Compensatory crosstalk between G-proteins is not an uncommon theme. For instance, Rap1 was originally identified in a search for proteins that reverse K-Ras induced transformation [51] and also the reciprocal actions of the related Rho-like G-proteins Rac1 and RhoA in endothelial permeability have been long established [52]. Compensation between Rap1 and Rap2 would explain why previous studies have observed only little [7], [9], [20] or no effect [53] of RapGAP overexpression on cell-cell adhesion. In fact, it has been suggested that endogenous RapGAP1 is required for the formation of cell-cell junctions, rather than counteracting it [18]. In line with this, depletion of RapGAP1 or RapGAP2 fails to increase the barrier resistance (Fig. 5E).

Despite the overlap in the regulation of Rap1 and Rap2, the data presented here have established Rap2 specific elements in the regulation of the barrier resistance. Rap2 depletion is phenocopied by depletion of the Rap2 specific GEF RasGEF1 and the Rap2 specific effector MAP4K4. It is likely that an additional element of regulation is added by the spatial organisation of Rap1 and Rap2 signalling. The overlap in Rap1 and Rap2 regulation on the one hand and the Rap2 specific elements on the other, would allow the tight and robust regulation of junctional permeability by two counteracting points of control access. What these access points are remains to be elucidated. For sure, both Rap1 and Rap2 require cadherins to regulate endothelial barrier, as depletion of Cadherins abolishes increased barrier resistance upon either Rap1 activation or Rap2 depletion. Hence, the opposite effects of Rap1 and Rap2 on barrier resistance are both likely relayed by Adherens Junctions. The Rap2 effector MAP4K4 has been shown to downregulate E-cadherin during mouse gastrulation [37]. However, we do not observe changes in VE-cadherin staining intensity upon Rap2 depletion. Alternatively, the Rap2 effector MAP4K4 has actin severing capacity [32]–[35]. Actin has been considered the driving force of Rap1-mediated junction tightening, as Rap1-induced junctional actin formation occurs in the absence of VE-cadherin [8], but lateral stabilization of VE-cadherin in the cell-cell junction by Rap1 does require the actin cytoskeleton [21]. However, actin modulation is unlikely to be the hub for the antagonistic function of Rap1 and Rap2, as no changes in actin organization were observed upon Rap2 depletion. Furthermore, simultaneous activation of Rap1 and Rap2 by depletion of RapGAP1 or RapGAP2 increases junctional actin, while leaving the barrier resistance unchanged. This means that Rap2 antagonizes Rap1 signalling downstream of junctional actin and that the formation of junctional actin can be uncoupled from the barrier resistance by activation of Rap2. Hence, our data establish that Rap1-induced formation of junctional actin is not directly causative to increased barrier resistance. Instead, the barrier resistance is controlled by the reciprocal effects of Rap1 and Rap2, of which only the former is mediated by the actin cytoskeleton.
